# Supplementary material for: The Antiviral Effects of Jasminin via Endogenous TNF-α and the Underlying TNF-α-Inducing Action
Source: Molecules. 2022 Feb 28;27(5):1598. doi: 10.3390/molecules27051598 (PMC8911969; doi:10.3390/molecules27051598)
Supplement: Supplementary file 1 [file molecules-27-01598-s001.zip › molecules-1604363-supplementary.pdf]

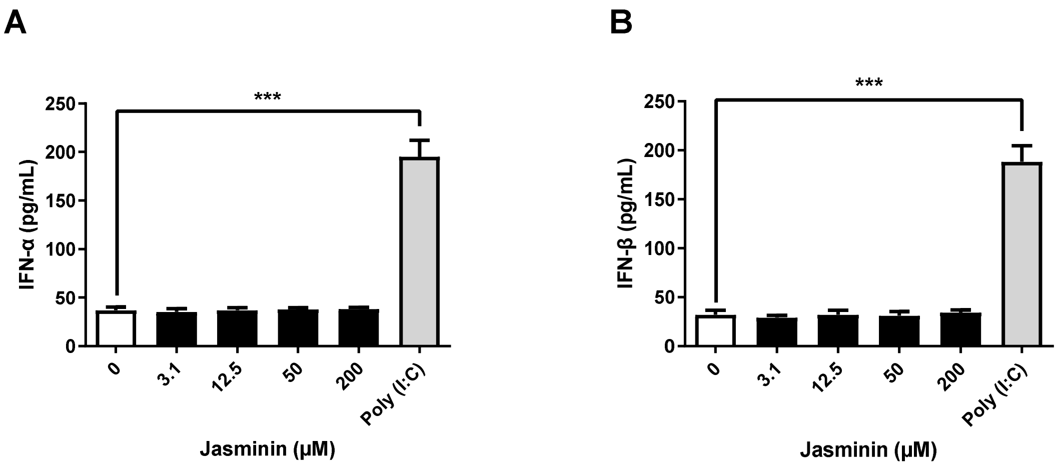

Figure S1. Effects of jasminin on expression of IFN-α and IFN-β in RAW 264.7 cells. RAW 264.7 cells were stimulated with jasminin (3.1, 12.5, 50, and 200 μM) for 24 h. IFN-α and IFN-β were measured by ELISA after the cell culture supernatants were harvested, and 0.25 μg/ml poly (I:C) was added as a positive control. ***p<0.001 Values are expressed as mean ± SD, n ≥ 3. * p<0.05, ** vs control (0: jasminin-untreated).

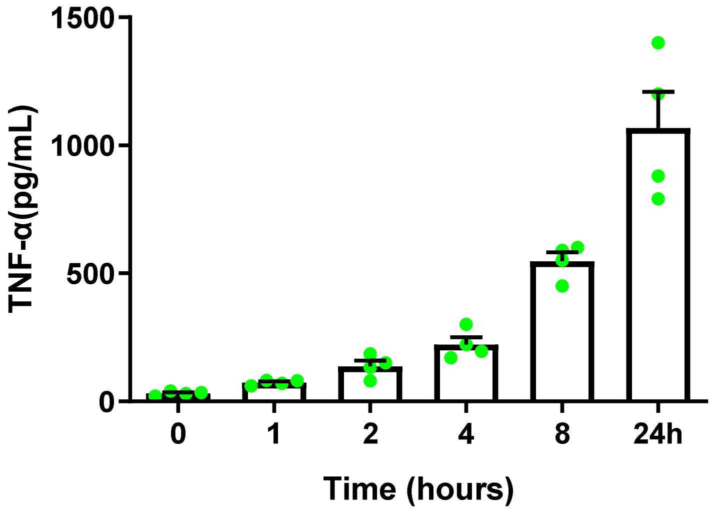

Figure S2. Kinetics of TNF-α production in RAW264.7 cells stimulated with jasminin (50 μM). Supernatants collected at different times were tested for the presence of TNF-α using a commercial ELISA kit. Means ± SD from four duplicate experiments.
